# Development and Preliminary Validation of a Central Venous Access Device‐Associated Skin Impairment Classification Tool Using Modified Delphi and Clinimetric Methods

**DOI:** 10.1111/jan.16416

**Published:** 2024-09-11

**Authors:** Hui (Grace) Xu, Jill Campbell, Mari Takashima, Emily Larsen, Fiona Coyer, Deanne August, Anna Dean, Colleen Pitt, Bronwyn Griffin, Nicole Marsh, Claire M. Rickard, Amanda Ullman

**Affiliations:** ^1^ Nursing and Midwifery Research Centre Royal Brisbane and Women's Hospital Herston, Brisbane Queensland Australia; ^2^ School of Nursing and Midwifery Queensland University of Technology Kelvin Grove, Brisbane Queensland Australia; ^3^ Schools of Nursing and Midwifery and Pharmacy and Medical Sciences, Alliance for Vascular Access Teaching and Research (AVATAR) Griffith University Brisbane Queensland Australia; ^4^ National Health and Medical Research Council Centre of Research Excellence (CRE) in Wiser Wound Care, Menzies Health Institute, Queensland Griffith University Nathan, Brisbane Queensland Australia; ^5^ School of Nursing, Midwifery and Social Work The University of Queensland Brisbane Queensland Australia; ^6^ Children's Health Queensland Hospital and Health Service Brisbane Queensland Australia; ^7^ School of Nursing and Midwifery Griffith University Brisbane Queensland Australia; ^8^ Herston Infectious Diseases Institute Metro North Health Herston, Brisbane Queensland Australia

**Keywords:** central venous access device, classification tool, definitions, Delphi method, nursing, skin impairment

## Abstract

**Background:**

An evidence and consensus‐based instrument is needed to classify central venous access device‐associated skin impairments.

**Aim:**

The aim of this study was to design and evaluate the central venous access device‐associated skin impairment classification tool.

**Design:**

A two‐phase modified Delphi study.

**Methods:**

This two‐phase study consisted of a literature review, followed by the development and validation of a classification instrument, by experts in the fields of central venous access devices and wound management (Phase 1). The instrument was tested (Phase 2) using 38 clinical photographs of a range of relevant skin impairments by the same expert panel. The expert panel consisted of registered nurses who were clinical researchers (*n* = 4) and clinical experts (*n* = 3) with an average of 24 years of nursing and research experience and 11 years of experience in wound management. Measures to assess preliminary content validity and inter‐rater reliability were used.

**Results:**

The instrument consists of five overarching aetiological classifications, including contact dermatitis, mechanical injury, infection, pressure injury and complex clinical presentation, with 14 associated subcategory diagnoses (e.g., allergic dermatitis, skin tear and local infection), with definitions and signs and symptoms. High agreement was achieved for preliminary scale content validity and item content validity (I‐CVI = 1). Inter‐rater reliability of aetiologies was high. The overall inter‐rater reliability of individual definitions and signs and symptoms had excellent agreement.

**Conclusion:**

The development and preliminary validation of this classification tool provide a common language to guide the classification and assessment of central venous access device‐associated skin impairment.

**Impact:**

The comprehensive and validated classification tool will promote accurate identification of central venous access device‐associated skin impairment by establishing a common language for healthcare providers. The availability of this tool can reduce clinical uncertainty, instances of misdiagnosis and the potential for mismanagement. Consequently, it will play a pivotal role in guiding clinical decision‐making, ultimately enhancing the quality of treatment and improving patient outcomes.

**Reporting Method:**

The Guidance on Conducting and Reporting Delphi Studies (CREDES) was adhered to.

**Patient or Public Contribution:**

No patient or public contribution.

## 1 Introduction

Central venous access devices (CVADs) are standard to support the treatment of many significant medical conditions. They provide an entry point to the central venous system to administer therapy that cannot be administered peripherally. It is estimated that 8% of adult hospital patients and 26% of paediatric patients require a CVAD during hospitalisation, with more than 5 million CVADs inserted in the United States annually (Liu et al. 2022; Ullman et al. 2017). Although vital in the delivery of medical therapy for patients with critical or complex health conditions, CVADs are associated with a range of complications, including CVAD‐associated skin impairment (CASI). The term CASI was first proposed by Broadhurst et al. (2017) and has now been incorporated into other clinical practice guidelines (Broadhurst et al. 2017; Gorski et al. 2021). These skin impairments include cutaneous infection, mechanical injuries such as skin tears or skin stripping, pressure injuries, contact dermatitis or a combination of these (Tian et al. 2021; Ullman, Kleidon, et al. 2019). However, accurate identification and classification of these complications can be challenging due to complex clinical presentations, clinician uncertainty and varying definitions of these skin injuries.

Accurate assessment and identification of CASI are essential, with diagnostic confusion impacting appropriate and timely treatment, and compromising patient care (Giardina et al. 2018). Classification of CASI relies on visual inspection, observation of clinical signs, assessor experience and patient‐reported symptoms (Broadhurst et al. 2017; Liu et al. 2022; Thayer 2012; Ullman et al. 2017) as well as an understanding of the possible aetiology of the presenting lesion. However, clinical presentation of CASI can be confusing, with several skin impairments with diverse aetiologies often coexisting. This complexity makes CASI classification difficult, often resulting in diagnostic and clinical uncertainty (Broadhurst et al. 2017; Liu et al. 2022). Therefore, the development of valid, reliable CASI definitions is vital to enable accurate assessment and differentiation of these complications.

## 2 Background

CASI is a significant and potentially avoidable form of patient harm, with 1.4%–12% of CVADs developing skin complications (Ullman, Kleidon, et al. 2019; Ullman, Mihala, et al. 2019). Many CVAD types exist, including nontunnelled, tunnelled and peripherally inserted central catheters and totally implantable venous access devices (Ullman, Cooke, et al. 2015), with the appropriate device chosen according to clinical need and clinical availability. CVAD use requires a break in skin integrity at the insertion site and the use of dressings and securement devices that themselves may further expose individuals to potential skin complications. Any individual with a CVAD is at risk of developing a skin impairment, particularly those who are extremely young (Ullman et al. 2017) or old (Farris et al. 2015), have frail skin (Beeckman et al. 2020; LeBlanc et al. 2021) or have multiple comorbidities (McNichol et al. 2013) at highest risk. Contributing factors for CASI can be multifactorial, intersecting and complex. These factors include trauma during insertion, irritants found in adhesives used in dressings or securement products, antiseptic cleaning agents or a combination of these products, excess moisture, mechanical forces such as skin stripping from tape or adhesive removal shear forces resulting from skin distention under unyielding dressings, or increased localised pressure from the device (Broadhurst et al. 2017; NPUAP, EPUAP, and PPIA 2019; Thayer 2012; Ullman, Marsh et al. 2015; Ullman, Kleidon, et al. 2019). Outcomes from CASI can be serious, burdensome for patients and clinicians and costly for health care providers.

Considerable efforts have been made to establish consistent definitions to classify individual and multiple components of CASI (Broadhurst et al. 2017; Thayer 2012; Ullman, Mihala, et al. 2019). Achieving consensus on a classification tool for CASI is important for multiple reasons. Firstly, accurate classification of skin complications depends on agreed, standardised definitions. Appropriate classification is pivotal in guiding evidence‐based clinical decision‐making, communication, reporting and outcome evaluation. Lack of an agreed language can contribute to clinical confusion, miscommunication and variability in care, ultimately impacting patient outcomes and safety (Jutel 2011; World Health Organization 2021). Secondly, a common language facilitates effective communication in regard to quality improvement, benchmarking and research (Jutel 2011; World Health Organization 2021). Thirdly, evidence‐based guidelines and protocols for clinical practice are underpinned by the imperative of a common language with which to define CASI (Jutel 2011; World Health Organization 2021). An agreed classification tool will guide clinical decision‐making, support treatment and prevention and improve patient safety. We aimed to address this evidence‐practice gap, by establishing consensus, validity and reliability of a new CASI classification tool.

## 3 The Study

### 3.1 Aims

This research aimed to (1) develop a CASI classification tool and (2) assess the evidence about its content validity and inter‐rater reliability.

## 4 Methods

### 4.1 Study Design

**FIGURE 1:**
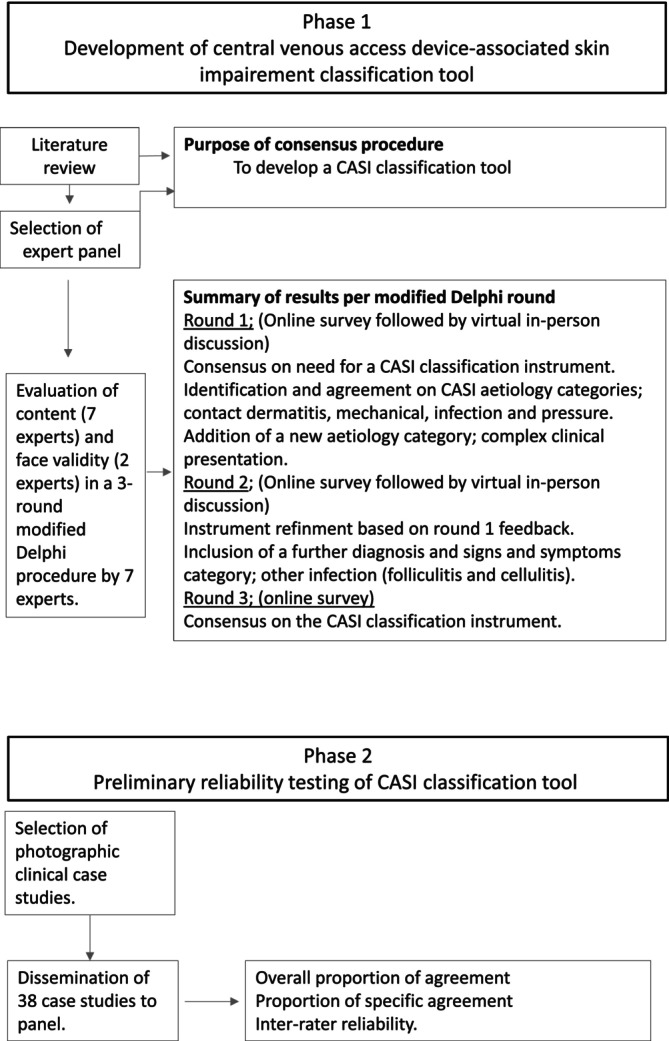
Process of design and preliminary validation of the CASI classification tool. CASI, central venous access device associated skin impairment.

This study utilised a Modified Delphi method to develop the CASI skin impairment classification tool, followed by preliminary clinimetric testing of validity and reliability in Queensland, Australia, from February 2022 to July 2023. An overview of the study procedure is presented in Figure 1. Study reporting has been informed by the Guidance on Conducting and Reporting Delphi Studies (CREDES) (Junger et al. 2017).

### 4.2 Study Procedure

#### 4.2.1 Phase 1—Development of CASI Classification System and Content Validation

This phase aimed to develop the CASI classification system and validate its contents. We began with a rapid review to swiftly screen and summarise relevant evidence concerning CASI classifications and terminologies. The lead author (GX) conducted a rapid review of the literature (Broadhurst et al. 2017; Fumarola et al. 2020; LeBlanc and Baranoski 2012; LeBlanc et al. 2013, 2018; McNichol et al. 2013; Thayer 2012; Ullman, Mihala, et al. 2019; van Tiggelen et al. 2020) and examined international guidelines (Gorski et al. 2021; Mermel et al. 2009; NPUAP, EPUAP, and PPIA 2019) related to CVAD use and CASI to identify relevant classifications and terminologies. We searched PubMed, CINAL and Embase databases using MeSH terms and the keywords ‘central venous catheters’, ‘central venous access devices’, ‘infection’, ‘medical adhesive‐related skin injury’, ‘skin tear’, ‘skin stripping’ ‘skin injury’, ‘contact dermatitis’ and ‘device‐related pressure injury’ between 2008 and 2022. Quantitative and qualitative studies published in English were included. Key international clinical practice guidelines, including the Infusion Therapy Standards of Practice (eighth edition) (Gorski et al. 2021), Clinical Practice Guidelines for the Diagnosis and Management of Intravascular Center‐Related Infection (2009) (Mermel et al. 2009) and Prevention and Treatment of Pressure Ulcers/Injuries: Clinical Practice Guideline (2019) (NPUAP, EPUAP, and PPIA 2019) were reviewed and included into the review. The literature review identified CASI classifications and terminology, with results compiled in tabular form for circulation to the panel (Table 1).

**TABLE 1:** Literature search results.

Authors	Aetiology identified	Diagnosis	Definition and signs and symptoms
Broadhurst et al. (2017)	Infection	Exit‐site infection	Characterised by redness, hardness and/or tenderness within 2 cm of catheter exit site; possible with other signs and symptoms of infection, such as fever or purulent drainage at exit site. Concomitant bloodstream infections may be present. Diagnosis should be confirmed via swab culture
	Skin injury	Skin stripping	Removal of one or more layers of the skin occurring following traumatic removal of adhesive tape or dressing. Lesions are frequently shallow and irregular in shape; skin may appear shiny or moist dark pink or red with significant discomfort if exposure to nerve endings. May have open lesions with erythema and blisters
		Skin tear	Wound caused by shear, friction and/or blunt force resulting in separation of skin layers (often related to traumatic dressing removal); can be partial or full thickness
		Tension blister	Wound (separation of the epidermis from the dermis) caused by shear force as a result of distention of skin under an unyielding adhesive tape or dressing; inappropriate strapping of tape dressing during application, or when a joint or other area of movement is covered with an unyielding tape
	Skin irritation	Irritant contact dermatitis	Nonallergic reaction to chemical irritant; well‐defined affected area correlates with the area of exposure; may be reddened and swollen and vesicles present; typically, of shorter duration
		Allergic contact dermatitis	Cell‐mediated immunologic response to a component of a product; typically, area of erythematous, vesicular, pruritic dermatitis corresponding to area of exposure and/or beyond, which may persist for up to a week. Irritant/allergen may be a component of the antiseptic solution, skin barrier solution or dressing
	Weeping/oozing (noninfectious)	Drainage at the CVAD site	Drainage at CVAD^a^ site. Clear amber; often considered normal (but may be associated with infection or lymph node nicked during insertion). Pink or red; Due to the presence of red blood cells; often related to trauma of CVAD insertion, particularly in neutropenic patients. Cloudy milky; may indicate fibrin strands (a response to inflammation) or infection (purulent exudates containing white blood cells or bacteria)
National Pressure Ulcer Advisory Panel (NPUAP), European Pressure Ulcer Advisory Panel (EPUAP), and PPIA (2019)	Device‐related pressure injury	Stage 1	Intact skin with nonblanchable redness of a localised area usually over a bony prominence. Darkly pigmented skin may not have visible blanching; its colour may differ from the surrounding areas. The area may be painful, firm, soft, warmer or cooler as compared to adjacent tissue
	Device‐related pressure injury	Stage 2	Partial‐thickness loss of dermis. Presents as a shallow open ulcer with a red pink wound bed, without slough. May also present as an intact or open/ruptured serum‐filled blister
	Device‐related pressure injury	Stage 3	Full‐thickness tissue loss. Subcutaneous fat may be visible, but bone, tendon or muscle are not exposed. Slough may be present but does not obscure the depth of tissue loss
	Device‐related pressure injury	Stage 4	Full‐thickness tissue loss with exposed bone, tendon or muscle. Slough or eschar may be present on some parts of the wound bed. Often include undermining and tunnelling. The depth of a stage IV pressure injury varies by anatomical location
	Device‐related pressure injury	Unstageable	Full‐thickness skin and tissue loss in which the base of the injury is covered by slough (yellow, tan, grey, green or brown) and/or eschar (tan, brown or black) in the wound bed. Until enough slough or eschar is removed to expose the base of the wound, the stage cannot be determined. Excludes pressure injury reclassified to stage III of IV after exposure/debridement. Slough (yellow, tan, grey, green or brown) and/or eschar (tan, brown or black) present in the wound bed
	Device‐related pressure injury	Deep tissue injury. Depth unknown	Purple or maroon localised area of discoloured intact skin or blood‐filled blister due to damage of underlying soft tissue from pressure and/or shear. The area may be preceded by tissue that is painful, firm, mushy, boggy, warmer or cooler as compared to adjacent tissue. Deep tissue injury may be difficult to detect in individuals with dark skin tones. Evolution may include a thin blister over a dark wound bed. The wound may further evolve and become covered by thin eschar. Evolution may be rapid exposing additional layers of tissue even with optimal treatment
Fumarola et al. (2020)	Mechanical	Skin stripping	Removal of one or more layers of the epidermis (top layer of the skin). Damage is often shallow and irregular in shape. The skin may appear shiny. Open sore may be accompanied by red skin and blister formation
		Tension injury or blister	Injury caused by shear force (separation of the epidermis from the dermis, which is the second layer of the skin)
		Skin tear	Skin is pulled away and the layers of the skin separate. This can cause either a partial‐thickness wound (one that extends into the epidermis and dermis) or a full‐thickness wound (one that extends into fat and muscle layers
	Dermatitis	Irritant contact dermatitis	The skin is inflamed (red), and can become blistered, dry, thickened and cracked. Injury caused by shear force (separation of the epidermis from the dermis, which is the second layer of the skin)
	Other	Maceration	Skin appears wrinkled and white/grey. Softening of skin increases its permeability and susceptibility to infection
		Folliculitis	Appears as small in inflamed elevations of skin around the hair follicle. These can present as papules (skin that has changed colour or texture) or pustules
Mermel et al. (2009)	Infection	Exit‐site infection	Exudate at catheter exit site yields a microorganism with or without concomitant bloodstream Infection Clinical; Erythema, induration and/or tenderness within 2 cm of the catheter exit site; may be associated with other signs and symptoms of infection, such as fever or purulent drainage emerging from the exit site, with or without concomitant bloodstream infection
Thayer (2012)	No aetiology identified by the author	Moisture‐associated skin damage	Three primary changes when skin is exposed to excessive hydration…increased permeability of the stratum corneum, an alkaline shift in skin pH and inflammation. Clinically the skin is moist, soft and changes in colour, becoming white or grey. The texture becomes soggy and with ongoing exposure the skin may crumble
	Contact dermatitis	Irritant Contact dermatitis	A nonallergic inflammatory response triggered by exposure to an irritating substance
		Allergic contact dermatitis	An immune‐mediated response
	Adhesive trauma	Skin stripping	Delamination of layers of the epidermis or detachment of the entire epidermis from the epidermis…repeated adhesive removal can easily sufficient layers… over time inflammation will be noted
		Tension blisters	Tension blisters form under adhesives… These are commonly unroofed with adhesive removal, leaving painful partial‐thickness wounds
	Infection	Folliculitis	An inflammation or infection of hair follicles and is characterised by erythema and pustules that form immediately around the follicles
		Cutaneous fungal infection	Candidiasis is often accompanied by diffuse erythema
Le Blanc et al. (2013)	Skin tear	Skin tear; Type 1—no skin loss	Linear or flap tear that can be repositioned to cover the wound bed
		Skin tear; Type 2—partial flap loss	Partial flap loss that cannot be repositioned to cover the wound bed
		Skin tear; Type 3—total flap loss	Total flap loss exposing entire wound bed
McNicol et al. (2013)	Mechanical	Skin (epidermal) stripping	Removal of one or more layers of the stratum corneum occurring following removal of adhesive tape or dressing; lesions are frequently shallow and irregular in shape and skin may appear shiny; open lesions may be accompanied by erythema and blister formation
		Tension injury or blister	Injury (separation of the epidermis from the dermis) caused by shear force as a result of distention of skin under an unyielding adhesive tape or dressing, inappropriate strapping of tape or dressing during application, or when a joint or other area of movement is covered with unyielding tape
		Skin tear	Wound caused by shear, friction and/or blunt force resulting in separation of skin layers; can be partial or full thickness
	Dermatitis	Irritant contact dermatitis	Nonallergic contact dermatitis occurring as a result of a chemical irritant; a well‐defined affected area correlates with the area of exposure; may appear reddened and swollen and vesicles may be present; typically, a shorter duration
		Allergic dermatitis	Cell‐mediated immunologic response to a component of tape adhesive or backing; typically appears as an area of exposure and/or beyond; persists for up to a week
	Other	Maceration	Changes in the skin resulting from moisture being trapped against the skin for a prolonged period; skin appears wrinkled and white/grey in colour; softening of the skin results in increased permeability and susceptibility to damage from friction and irritants
		Folliculitis	Inflammatory reaction in hair follicle caused by shaving or entrapment of bacteria; appears as small inflamed elevations of skin surrounding the hair follicle; may be nonsuppurative (papules) or contain pus (pustules)
Ullman, Mihala, et al. (2019)	No aetiology identified by the author	Bruise	Not defined by author
	No aetiology identified by the author	Infiltration	Involves localised swelling
	No aetiology identified by the author	Dermatitis	Raised red rash, with or without vesicles, which persisted for greater than 30 min
	Mechanical injury	Skin tear	Not defined by author
	No aetiology identified by the author	Blister	Not defined by author
	Local infection	Local infection	Purulent discharge or redness extending 1 cm beyond the site that prompted clinicians to order device removal, or commence antimicrobial therapy
	Vascular access‐associated individual skin signs or symptoms	Fluid leakage from vascular access site	Not defined by author
		Erythema from vascular access site	Not defined by author
		Pain from vascular access site	Not defined by author
		Itch from vascular access site	Not defined by author
van Tiggelen et al. (2020)	Skin tear	Skin tear; Type 1—no skin loss	Linear or flap tear that can be repositioned to cover the wound bed
		Skin tear; Type 2—partial flap loss	Partial flap loss that cannot be repositioned to cover the wound bed
		Skin tear; Type 3—total flap loss	Total flap loss exposing entire wound bed

Abbreviation: CVAD, central venous access device.

The expert panel was selected by lead and senior authors (GX and AU). Seven experts were selected using purposive sampling within the authors' networks, with selection based on research and clinical expertise in the fields of CVAD use, tool development, infection prevention, skin integrity and wound care, including representation across differing age groups (neonates, paediatric and adults). Several of these individuals (AU, FC, JC, CR) are experts on clinimetric studies and have experience in tool development (Barakat‐Johnson et al. 2022; Broadhurst et al. 2017; Schults et al. 2021, 2022; Ullman et al. 2020). The invitation to participate was sent via email to eight clinical or research experts to achieve acceptable CVI values of at least 0.83 (Lynn 1986; Polit, Beck, and Owen 2007). Seven agreed to participate as panel members and one declined. An online survey was developed (LimeSurvey https://www.limesurvey.org) to enable feedback on the domains of CASI aetiology, definitions and signs and symptoms.

A modified Delphi method was employed, incorporating virtual in‐person discussions to facilitate deeper exploration of complex issues and promote rapid consensus building among experts (Trevelyan and Robinson 2015). This is considered a modified Delphi with the inclusion of virtual in‐person discussions in each round (Keeney, McKenna, and Hasson 2011). The purpose of the three‐round Delphi method is to achieve consensus among the expert panel regarding the development of a need for a CASI classification tool, content and categories of the tool. Building on the findings of the literature review, the first round established the need for the development of a CASI consensus instrument and achieved broad agreement on the content and categories for inclusion. The panel was asked to review and rate a list of CASI classifications and terminology using a standard content validation form for relevance and appropriateness. In Round 2, the goal was to refine and improve the proposed instrument. Panel members were requested to revise the CASI classifications and terminology, providing comments and suggestions. Round 3 aimed to further refine the instrument based on the previous two rounds and reach a consensus on the final instrument. During this round, a revised list of CASI classifications and terminology, based on suggestions from Round 2, was sent to the panel members. They were given another opportunity to agree, disagree or propose further revisions to the CASI classifications and terminology.

In short, three survey rounds were conducted, including two virtual meetings using an online meeting platform (Microsoft Teams https://www.microsoft.com/en‐AU/microsoft‐teams) to discuss the need for consensus on a CASI classification tool and allow the panel to resolve any questions on the proposed CASI aetiology, definitions and signs and symptoms. The panel was invited to provide structured feedback individually and independently, in the domains of aetiology and diagnosis relevance, definition appropriateness and symptom appropriateness using a 4‐point ordinal scale (1 = not relevant, 2 = somewhat relevant, 3 = quite relevant, 4 = highly relevant), with feedback to either delete, revise or keep individual criteria. The panellists were able to provide additional comments for each category. The purpose of the meeting was to allow panellists to discuss the content of the tool and clarify any questions. At the conclusion of the validation process, the agreed definitions were revised and sent to the panel for a final round to achieve consensus on the CASI classification tool before moving to Phase 2.

#### 4.2.2 Phase 2—Reliability Testing of the CASI Classification System

The aim of this phase was to examine inter‐rater reliability of the proposed classification tool developed in Phase 1 and consisted of two steps.

The first step was the selection of clinical images (Figure 1) and the development of an online survey Clinical images of a variety of CASI were selected from a multisite, randomised controlled trial (RCT) conducted between 2017 and 2020 in Queensland, Australia (Rickard et al. 2017). Patient (or representative) consent had been gained to use these clinical images accompanied by data on patient age, gender, body mass index, primary diagnosis, device type and date inserted, date of image, clinical signs, patient‐reported symptoms and microbiology results if applicable (Rickard et al. 2017). The RCT participants were cancer patients being treated in two tertiary hospitals in Queensland, including one paediatric hospital. The images were purposively selected to equally represent the agreed CASI categories. The images were selected by researchers (GX and EL), who are experts in CVAD use and CASI. The original images were captured by the research assistants during the source RCT, who had received education on how to achieve consistent quality clinical images. Case studies were excluded if the clinical images were sub‐optimal (e.g., images were out of focus or the insertion site was not visible). We used cases instead of observations to prevent the selection of the same cases/patients.

To calculate case sample size, we assumed the expected inter‐rater reliability (intra‐class coefficient) to be 0.75 with a precision of ±0.10 (alpha = 0.05), resulting in a sample of 38 cases with images required with a minimum of 6 raters (Bonett 2002). The case studies were rated according to the agreed upon CASI aetiology, definitions using a 5‐point ordinal scale (1 = strongly agree, 2 = agree, 3 = undecided, 4 = disagree, 5 = strongly disagree) (Mokkink et al. 2010). A pilot test of two case studies was circulated to two panel reviewers for face validity to ensure that reviewers have a good understanding of the reliability form and selected cases. Inter‐rater reliability was calculated to measure variability and differences between the reviewers in the expert panel (Kottner et al. 2011). The 38 clinical images were selected to provide accurate and diverse representations of CASI in the aetiology and diagnosis categories of the instrument. These images represented CASI from the aetiology category of dermatitis (10 images), mechanical injury (7 images), infection (4 images), pressure injury (3 images) and complex clinical presentation (14 images). In addition, all these selected cases were reviewed by two panel members before circulating them to the entire expert group in the following step.

The second step was to circulate a survey to the same expert panel as Phase 1, via email with a link to an online survey (software package LimeSurvey https://www.limesurvey.org), who were asked to classify the case studies according to the agreed CASI aetiology, definitions and signs and symptoms developed in Phase 1. Additionally, experts were provided a PowerPoint file containing slides for each of the 38 case studies. The case studies and associated images were representative of the categories in the tool. Each case study slide included one or more images of the device insertion site, associated CASI and clinical details (patient age, gender, body mass index, primary diagnosis, device type and date inserted, date of the image, clinical signs of the CASI, patient‐reported symptoms related to the CASI and microbiology results relevant to the device (e.g., *Corynebacterium jeikeium*)), Mihala, for the respective patient. Based on clinical images and information in this PowerPoint, the expert panel was required to use the tool to classify the CASI. Following the initial case study review round, a virtual panel meeting was held for the panellists to discuss the results of the survey and amend the classification system if required. A final round was circulated to the expert panel using the agreed CASI definitions via an online survey (software package LimeSurvey https://www.limesurvey.org) to establish the IRR of the proposed CASI classification tool.

### 4.3 Ethical Considerations

Ethical approval was obtained from the Children's Health Queensland Hospital and Health Service Human Research Ethics Committee (HREC/15/QRCH/241) and Griffith University Ethics Committee (2016/063). All participants received full information prior to the commencement of the study. Consent to participate was assumed with the return of the completed questionnaire.

### 4.4 Data Analysis

Data management and analysis were conducted by using Stata 14 (StataCorp., 2015, College Station, TX: StatCorp LP). Descriptive statistics were used to describe the validation results, including frequency, percentages, means and standard deviations.

#### 4.4.1 Content Validity

The preliminary content validity index (CVI) for each category in Phase 1 was calculated: item‐CVI (I‐CVI) was calculated by dividing the number of experts providing a score of 3 or 4 divided by the total number of experts, scale‐CVI (S‐CVI/Ave; scale‐level content validity index based on the average method) based on average was calculated by the sum of item‐CVI divided by the number of items, and scale‐level content validity index based on the universal agreement method (S‐CVI/UA) based on the proportion of items on the scale that achieve a relevance scale of 3 or 4 by all experts (Yusoff 2019). CVI cut‐off was set at 0.83 with six to eight experts (Yusoff 2019).

#### 4.4.2 Reliability

To assess the inter‐rater reliability of the CASI classification system in Phase 2, Fleiss' Kappa was used to assess the reliability of agreement between multiple raters. In the event of an extremely high agreement between the raters leading to kappa paradox, a modified Kappa or Gwet's AC2 value was used (Zec et al. 2017). We used the evaluation criteria that are proposed by Ciccetti and Sparrow (1981): Fair = k* of 0.40–0.59; Good = k* of 0.60–0.74; and Excellent = k* of 0.75–1.00.

## 5 Results

### 5.1 Phase 1 Results

#### 5.1.1 Literature Review and Development of CASI Classification Tool

CASI aetiology categories, definitions and associated signs and symptoms were identified from the literature (Table 1). A survey that presented a summary of the literature review proposing four CASI aetiology categories and 13 definitions and associated signs and symptoms was presented to the expert panel via email.

#### 5.1.2 Panellist Expert Characteristics

The expert panel consisted of seven specialist nurses from Queensland Australia, with diverse clinical and research experience (Table S1). Consisting of seven registered nurses, the panel included four clinical researchers (57%) and three clinical experts (43%), with an average of 24 years (SD = 12.43) of nursing experience and an average of 11 years (SD = 4) of expertise in wound management. Each panel member possesses advanced qualifications and expertise in areas such as wounds and skin integrity, vascular access and/or infection prevention, across neonatal to geriatric care.

#### 5.1.3 Content Validity of CASI Classification Tool

The initial panel meeting began with a panel discussion that identified the need for consensus regarding CASI definitions. There was unanimous acknowledgement that an agreed classification tool would improve clinical outcomes by reducing diagnostic confusion, enhance clinical documentation and communication and provide a common language to inform quality improvement, benchmarking and research. The panel suggested that the classification framework should include overarching aetiology classifications, with associated subcategories of diagnoses, definitions, and signs and symptoms. This is consistent with existing literature and supports diagnosis and clinical decision‐making (Broadhurst et al. 2017; LeBlanc et al. 2013).

Following the initial virtual meeting and online survey, the panel identified the challenge of classifying a CASI where there may be multiple or indeterminate aetiological factors for a presenting skin impairment. To address this gap, the panel proposed a new aetiology category; complex clinical presentation, thereby improving the accuracy and comprehensiveness of CASI classification. They agreed that this category reflected the complex diagnostic challenge that CASI presents. Additionally, the panel suggested two diagnostic subcategories be added to types of infection: folliculitis and cellulitis as these infections are sometimes observed at CVAD sites yet were not consistently identified in the literature (Table 2). Overall, experts supported the proposal, and discrepancies concerning the nomenclature of the aetiology, CASI diagnosis, definitions, and signs and symptoms were resolved through group discussion, leading to consensus. No conflicting opinions or disagreements arose.

**TABLE 2:** The agreed central venous access device‐associated skin impairment classification tool.

Aetiology	CASI* Diagnosis		CASI* Definition	CASI* Signs and symptoms	Original definition reference(s)
Dermatitis	Contact dermatitis		Nonallergic reaction to chemical irritants.	Well‐defined affected area correlates with the area of exposure; may be reddened and swollen and vesicles present; typically of shorter duration.	Broadhurst et al. (2017)
	Allergic dermatitis		Cell‐mediated immunologic response to a component of a product (e.g., Chlorhexidine gluconate decontaminant and adhesive).	Typically area of erythematous, vesicular, pruritic dermatitis corresponding (initially) to an area of exposure and/or beyond, which may persist for up to a week.	Broadhurst et al. (2017)
Mechanical injury	Skin stripping		Separation or removal of one or more layers of the stratum corneum (outer layer of the epidermis or skin) occurring following removal of adhesives, tapes or dressing.	Lesions are frequently shallow and irregular in shape; skin may appear shiny or moist, dark pink or red with discomfort if exposed to nerve endings. May present as open lesions with erythema and/or blisters (intact or nonintact).	Broadhurst et al. (2017)
	Skin tears		A traumatic wound caused by mechanical forces, including removal of adhesives. Severity may vary by depth (not extending through the subcutaneous layer).	Visible separation of skin layers with underlying tissue exposed (dark pink or red in appearance), often with associated discomfort or pain. Skin tears are classified as type 1 no skin loss, type 2 partial skin/flap loss and type 3 total flap loss.	LeBlanc et al. (2018)
	Tension injury		Separation of the epidermal layers (e.g., epidermis from the dermis) caused by shear force. This can be caused as a result of distension of skin under an adhesive, tape or dressing; inappropriate strapping of tape or dressing during application, often when a joint or other area of movement is covered with an unyielding tape.	May present as intact blisters (with serum or blood‐filled layer) or nonintact (burst fluid‐filled layer) blisters. Often associated with discomfort or pain, especially on further contact.	Broadhurst et al. (2017)
Infection	Local infection		Symptomatic insertion site with organism(s) from skin or purulent fluid swab identified by a culture or nonculture based microbiologic testing method, which is performed for purposes of clinical diagnosis, or treatment catheter exit site yields a microorganism; OR other identifiers of local infection in accordance with National Health and Safety Network^a^ (e.g., symptomatic patient, with a positive tip culture [>15 cfu]). Can occur with or without concomitant bloodstream infection.	Erythema, induration, heat and/or tenderness at/near the catheter exit site; may be associated with other signs and symptoms of infection, such as fever or purulent drainage emerging from the exit site.	Mermel et al. (2009) and European Pressure Ulcer Advisory Panel, National Pressure Injury Advisory Panel and Pan Pacific Pressure Injury Alliance (2019)
	Other type of skin infection		Infection of the skin within the perimeter of the dressing, but not at the insertion site. Can include folliculitis, cellulitis, etc.	Individual signs and symptoms may vary depending on the type of skin infection.	
Pressure injury	Medical device‐related pressure injuries	Result from the use of devices designed and applied for diagnostic and therapeutic purposes and nonmedical devices that inadvertently remain in contact with the skin. The resultant pressure injury generally conforms to the pattern of the device as a result of localised damage from the device to the skin and/or underlying tissue, as a result of pressure or pressure in combination with shear.		As per the categorisation below.	National Pressure Ulcer Advisory Panel (NPUAP), European Pressure Ulcer Advisory Panel (EPUAP), and PPIA (2019)
		Stage I pressure injury	Intact skin with nonblanchable redness of a localised area usually over a bony prominence.	Darkly pigmented skin may not have visible blanching; its colour may differ from the surrounding areas. The area may be painful, firm, soft, warmer or cooler as compared to adjacent tissue.	
		Stage II pressure injury	Partial‐thickness loss of dermis.	Presents as a shallow open ulcer with a red pink wound bed, without slough. May also present as an intact or open/ruptured serum‐filled blister.	
		Stage III pressure injury	Full‐thickness tissue loss.	Subcutaneous fat may be visible, but bone, tendon or muscle are not exposed. Slough may be present but does not obscure the depth of tissue loss.	
		Stage IV pressure injury	Full‐thickness tissue loss with exposed bone, tendon or muscle.	Slough or eschar may be present on some parts of the wound bed. Often include undermining and tunnelling. The depth of a stage IV pressure injury varies by anatomical location.	
		Unstageable pressure injury	Full‐thickness skin and tissue loss in which the base of the injury is covered by slough (yellow, tan, grey, green or brown) and/or eschar (tan, brown or black) in the wound bed. Until enough slough or eschar is removed to expose the base of the wound, the stage cannot be determined. Excludes pressure injury reclassified to stage III of IV after exposure/debridement.	Slough (yellow, tan, grey, green or brown) and/or eschar (tan, brown or black) present in the wound bed.	
		Suspected deep tissue injury, depth unknown	Purple or maroon localised area of discoloured intact skin or blood‐filled blister due to damage of underlying soft tissue from pressure and/or shear.	The area may be preceded by tissue that is painful, firm, mushy, boggy, warmer or cooler as compared to adjacent tissue. Deep tissue injury may be difficult to detect in individuals with dark skin tones. Evolution may include a thin blister over a dark wound bed. The wound may further evolve and become covered by thin eschar. Evolution may be rapid exposing additional layers of tissue even with optimal treatment.	
Complex clinical presentation	Complex clinical presentation		More than one aetiology and presentation and may include device‐related pressure injury, mechanical injury, immunology‐medicated reaction (e.g., dermatitis) or infection within the same CVAD site. May be difficult to determine primary and specific aetiology.	Individual signs and symptoms as above. These may overlap in symptomatology due to complex and interacting aetiologies.	

Abbreviation: CVAD, central venous access device.

^a^ Central venous access device‐associated skin impairment classification.

The final proposed CASI classification tool contains five aetiological classifications, with 14 definitions and signs and symptoms subcategories (Table 2). The CVI for all aetiology classifications were (I‐CVI = 1), and the scale was (S‐CVI/Ave = 1 and S‐CVI/UA = 1) and met a satisfactory level with a high agreement of the preliminary content validity (Table 3). The CVI was also 1 for all individual definitions and signs and symptoms (I‐CVI = 1) and the scale (S‐CVI/Ave = 1 and S‐CVI/UA = 1) and had a satisfactory high level of agreement for the preliminary content validity (overall kappa 0.95, Table 4).

**TABLE 3:** Content validity of aetiology definitions.

	Number of raters	Relevance	Appropriateness—definition	Item recommendation	Appropriateness—signs and symptoms	Experts in agreement	I‐CVI	UA
		Number of raters who rated 3 or 4						
Contact dermatitis	7	7	7	7	7	7	1	1
Mechanical injuries	7	7	7	7	7	7	1	1
Local infection	7	7	7	7	7	7	1	1
Device‐related pressure injury	7	7	7	7	7	7	1	1
Complex combination	7	7	7	7	7	7	1	1
						S‐CVI/Ave		1
Proportion relevance		1	1	1	1	S‐CVI/UA		1

*Note:* Scoring 1 = not relevant; 2 = somewhat relevant; 3 = quite relevant; 4 = highly relevant.

Abbreviations: I‐CVI, item‐level content validity index; S‐CVI/Ave, scale‐level content validity index based on the average of I‐CVI scores; S‐CVI/UA, scale‐level content validity index based on the universal agreement method.

**TABLE 4:** Content validity of individual definitions and signs and symptoms.

	Number of raters	Relevance	Appropriateness—definition	Item recommendation	Appropriateness—signs and symptoms	Experts in agreement	I‐CVI	UA
		Number of raters who rated 3 or 4						
Irritant contact dermatitis	7	7	7	7	7	7	1	1
Allergic contact dermatitis	7	7	7	7	7	7	1	1
Skin stripping	7	7	7	7	7	7	1	1
Skin tears	7	7	7	7	7	7	1	1
Tension blisters	7	7	7	7	7	7	1	1
Local infection	7	7	7	7	7	7	1	1
Other infection (e.g., folliculitis, cellulitis)	7	7	7	7	7	7	1	1
Device‐related pressure injury, overall	6	6	6	6	6	6	1	1
Device‐related pressure injury, stage I	7	7	7	7	7	7	1	1
Device‐related pressure injury, stage II	7	7	7	7	7	7	1	1
Device‐related pressure injury, stage III	7	7	7	7	7	7	1	1
Device‐related pressure injury, stage IV	7	7	7	7	7	7	1	1
Device‐related pressure injury, unstageable	7	7	7	7	7	7	1	1
Device‐related suspected deep tissue injury, depth unknown	7	7	7	7	7	7	1	1
Complex clinical presentation	7	7	7	7	7	7	1	1
						S‐CVI/Ave		1
Proportion relevance		1	1	1	1	S‐CVI/UA		1

*Note:* Scoring 1 = not relevant; 2 = somewhat relevant; 3 = quite relevant; 4 = highly relevant.

Abbreviations: I‐CVI, item‐level content validity index; S‐CVI/Ave, scale‐level content validity index based on the average of I‐CVI scores; S‐CVI/UA, scale‐level content validity index based on the universal agreement method.

### 5.2 Phase 2 Results

#### 5.2.1 Inter‐rater Reliability of CASI Classification Tool

In Phase 2, the overall inter‐rater reliability of aetiological classifications exceeded 0.9 Gwet's AC2, except for contact dermatitis (Gwet's AC2 = 0.87) (Table 5).

**TABLE 5:** Inter‐rater reliability for aetiology classifications.

Aetiology classification	Percent agreement	Percent chance agreement	Gwet's AC2	95% low	95% high
Contact dermatitis	0.88	0.01	0.87	0.37	1.00
Mechanical injuries	0.94	0.01	0.94	0.64	1.00
Infection	1.00	0	1.00	NA^a^	NA^a^
Device‐related pressure injury	1.00	0	1.00	NA^a^	NA^a^
Complex clinical presentation	0.96	0.09	0.96	0.91	0.99

*Note:* We used the evaluation criteria that are proposed by Ciccetti and Sparrow (1981): Fair = k* of 0.40–0.59; Good = k* of 0.60–0.74; and Excellent = k* of 0.75–1.00.

^a^ Due to complete agreement.

The overall inter‐rater reliability of individual definitions and signs and symptoms had excellent agreement (kappa coefficient 0.95, *z* = 50.96, *p* < 0.01) (Table 6). Allergic dermatitis had the lowest agreement, with the kappa coefficient of 0.76 (*z* = 18.10, *p* < 0.01) (Table 6).

**TABLE 6:** Inter‐rater reliability of individual definitions and signs/symptoms.

Outcome	Kappa	*z*	Prob>*Z*
Irritant contact dermatitis (1)	0.97	23.23	<0.01
Allergic contact dermatitis (2)	0.76	18.10	<0.01
Skin stripping (3)	1.00	23.87	<0.01
Skin tears (4)	1.00	23.87	<0.01
Tension blisters (5)	1.00	23.87	<0.01
Local infection (6)	0.94	22.36	<0.01
Other infection (e.g., folliculitis, cellulitis) (14)	1.00	23.87	<0.01
Device‐related pressure injury, stage I (7)	1.00	23.87	<0.01
Device‐related pressure injury, stage II (8)	1.00	23.87	<0.01
Device‐related pressure injury, stage III (9)	—	—	—
Device‐related pressure injury, stage IV (10)	—	—	—
Device‐related pressure injury, unstageable (11)	—	—	—
Device‐related suspected deep tissue injury, depth unknown (12)	—	—	—
Complex clinical presentation (13)	0.93	22.24	<0.01
Overall	0.95	50.96	<0.01

*Note:* We used the evaluation criteria that are proposed by Ciccetti and Sparrow (1981): Fair = k* of 0.40–0.59; Good = k* of 0.60–0.74; and Excellent = k* of 0.75–1.00.

## 6 Discussion

CVADs are used across healthcare settings but are associated CASI which is a prevalent and potentially preventable injury with substantial potential consequences, including pain and CVAD failure (Tian et al. 2021; Ullman, Cooke, et al. 2015). However, due to clinical and diagnostic uncertainty and complexity, CASI is often under‐recognised, misdiagnosed, unreliably reported and therefore poorly managed. We propose a CASI classification system with high scale (S‐CVI = 1.0) and item content (I‐CVI = 1.0) validity, that identifies five aetiology categories, with 14 subcategories that define specific CASI and their associated signs and symptoms (Table 2). We have provided reliability data to support this new classification system, using photographic case studies by experts representing diverse nursing specialties. High inter‐rater reliability was demonstrated, with kappa coefficients of 0.76–1.00 across aetiology, definitions and associated signs and symptoms, indicating excellent agreement (Ciccetti and Sparrow 1981). Our classification system thus provides a new validated, comprehensive framework to support assessment, categorisation and clinical decision‐making, while providing a standardised language to healthcare providers and researchers.

To date, nine publications have directly identified and defined CASI complications (Table 1). The Infectious Diseases Society of America (2009) identifies intravascular catheter‐related exit‐site infection as a potential CASI (Mermel et al. 2009). The International Wound Infection Institute (2022) identified skin tears, pressure injury and infection (Swanson et al. 2022). Thayer's seminal work (2012) identifies four aetiology categories including moisture‐associated skin damage, contact dermatitis, adhesive trauma and infection (Thayer 2012). Associated with the aetiology category were six CASI subcategories including moisture‐associated skin damage, irritant and allergic contact dermatitis, folliculitis and cutaneous fungal infection. Broadhurst et al. (2017) developed and validated an algorithm to improve the identification and treatment of CASI in the categories of skin injury, exit‐site infection, noninfectious exudate and skin irritation (Broadhurst et al. 2017). These terms were defined and validated, with associated signs and symptoms presented. A secondary analysis of 13 studies (10,859 devices) by Ullman, Mihala, et al. (2019) identified six CASI complications which included bruise, infiltration, dermatitis, mechanical injury (tear), mechanical injury (blister), local infection and four common signs and symptoms (Ullman, Mihala, et al. 2019). A literature review by Duwadi, Zhao, and Budal (2019) classified CASI according to severity, with major complications including infection, thrombus formation and mechanical failure (Duwadi, Zhao, and Budal 2019). This study identifies skin reactions such as phlebitis, skin reactions to dressings, pain and bruising as common but minor complications. The primary objective of these studies was to identify skin complications associated with CVAD use, and to provide support for clinical practice, whereas we have developed a validated tool for CASI classification.

Our proposed CASI classification tool (Table 2) based on literature (Broadhurst et al. 2017; Mermel et al. 2009; Thayer 2012; Ullman et al. 2017) and expert consensus and tested with photographs selected from real clinical cases studies. The use of clinical photographs is consistent with research in this field (Beeckman et al. 2018; van Tiggelen et al. 2020) and contributes a validated framework to guide diagnosis, support practice and provide a common language for healthcare providers, educators and researchers. In addition, our tool is informed by internationally validated categorisation systems for skin impairments that can occur either with CVAD use, or independently of CVAD use, and have been included in our tool. These include pressure injury (NPUAP, EPUAP, and PPIA 2019), skin tears (LeBlanc and Baranoski 2012; LeBlanc et al. 2013) and medical adhesive‐related skin injury (Fumarola et al. 2020; McNichol et al. 2013). The international pressure injury categorisation system (NPUAP, EPUAP, and PPIA 2019) and the international skin tear classification system (LeBlanc and Baranoski 2012; LeBlanc et al. 2013; van Tiggelen et al. 2020) provide skin injury severity ratings that are determined by the degree of tissue loss and/or the type of tissue present in the wounds. While these severity ratings are included in our tool, they are specific to pressure injuries or skin tears resulting from CVAD use. The severity ratings associated with pressure injury and skin tears do not apply to CASI of other aetiology, nor does our tool identify overall CASI severity or degree of tissue loss. The clinical complexity of CASI can present significant classification challenges.

Our comprehensive tool recognises the imperative of understanding CASI aetiology by identifying five aetiological categories that will reduce diagnostic confusion and support decision‐making. Determining wound or skin injury aetiology is a critical first step in CASI assessment and will guide subsequent clinical decision‐making. Determining wound or skin injury aetiology is recognised as an essential component of wound care and is consistent with best practice in this field (Ayello 2005; Carville 2017; NPUAP, EPUAP, and PPIA 2019). Determining CASI aetiology can be complex due to the often multifactorial, intersecting or interacting aetiological factors. These factors may include the mechanical forces of skin stripping or skin tension, reaction to the adhesives in the dressing, infection or increased localised pressure and/or shear. It is not uncommon for several of these factors to be present concurrently, contributing to diagnostic confusion, delays in management decisions and compounding skin vulnerability to injury. As a result, the panel agreed that the category of complex clinical presentation be added to the tool. This category is applicable where more than one aetiology is present, or where the primary CASI aetiology cannot be determined. The panel also agreed that the inclusion of another diagnosis subcategory ‘other type of skin infection’ under the infection aetiology category was required to reflect the other potential infections such as folliculitis or cellulitis that may be associated with CVAD use. After a three‐round modified Delphi process, consensus was achieved on the definitions and addition of these new categories. These important additions contribute to our detailed and comprehensive classification tool.

Inter‐rater reliability was excellent for overall and item level, with high kappa values for almost all items from a clinimetric perspective, according to the proposed interpretation by Ciccetti and Sparrow (1981). Reliability of the tool was tested by the expert panel using clinical photographic case studies. However, in real‐world clinical practice, in addition to visual inspection CASI diagnosis would be supported by palpation, microbiological testing and eliciting patient‐reported symptoms such as pain, purulent discharge or pruritis. The panel agreed that some diagnostic challenges arose from the two‐dimensional nature of the photographs used in the survey. However, clinical photographs have been used successfully to rate the reliability of an instrument in several studies in the skin integrity field (Beeckman et al. 2018; Carville et al. 2022; van Tiggelen et al. 2020).

Our study revealed commonalities in CASI terminology in the literature, with recognition of the aetiological factors infection, contact dermatitis or mechanical forces (resulting in skin tears or blisters) (Broadhurst et al. 2017; Mermel et al. 2009; Thayer 2012; Ullman et al. 2017). However, the aetiological category of contact dermatitis is the subject of linguistic confusion. Synonyms for this term in the literature include skin irritation (Broadhurst et al. 2017), skin reactions to dressings (Duwadi, Zhao, and Budal 2019) or dermatitis (Ullman et al. 2017). In the broader skin integrity literature, Carville et al. (2022) identify eight different definitions for contact dermatitis (Carville et al. 2022), exacerbating clinical and linguistic uncertainty. Our panel rated the term contact dermatitis as highly relevant (Table 3) and is consistent with the use of the term by Thayer (2012) and Broadhurst et al. (2017). The specific CASI diagnoses irritant and allergic contact dermatitis and associated signs and symptoms, achieved excellent inter‐rater reliability (Table 6). While achieving excellent agreement, the diagnosis allergic contact dermatitis had the lowest kappa coefficient (kappa = 0.76, *z* = 18.10, *p* < 0.01). The reason for this may be in part due to the complexity of that CASI, or the confusion surrounding contact dermatitis definitions in the literature. Our tool is a significant step forward in the systematic classification of CASI in clinical practice, education and research.

### 6.1 Limitations

To test reliability, the study used clinical photographs of CASI in cancer patients in acute care settings. These patients may have preexisting skin conditions due to cancer treatment and other comorbidities, and results may not be generalisable to clinical contexts. Secondly, despite our efforts, no photographs of patients with darker skin tones were available. It is accepted that visual assessment of darker skin tones is subject to bias and confusion (Gunowa 2022). Any future research should include images of CASI in patients with darker skin tones. Experts from this study represented qualifications and skills across neonatal to geriatric wounds and skin integrity, vascular access and/or infection prevention; no cases of CASI were available to represent neonates or younger paediatrics. In the absence of population specific studies, authors acknowledge this study can be used as a framework for further study including paediatric and neonatal case studies. Finally, the expert panellists, all from Southeast Queensland Australia, developed and tested the reliability of the classification system. The same expert panel was used to develop the instrument and to conduct preliminary validity and reliability testing. Our expert panel is comprised of individuals with extensive expertise in the field. Utilising the same panel for the development and evaluation of the instrument ensured continuity and consistency in understanding the research aim through the development and testing process. The panel's contextual knowledge is important in refining item wording, clarity and relevance, and providing nuanced feedback during the evaluation and preliminary content and reliability validation phase. In addition, leveraging the same panel optimised resources. We acknowledge this as a limitation with a potential for bias and recommend further external validity testing with a larger sample of potential users of the instrument. Additionally, we recommend further research to evaluate validity, reliability and generalisability, using participants from a variety of clinical contexts, experiences, knowledge and geographic locations.

### 6.2 Implications for Practice and Future Research

Recognising and diagnosing CASI‐related injuries can be challenging. Diagnostic accuracy is a complex process that requires consideration of various factors, particularly clinical judgement and extensive experience of individual clinicians (McNichol et al. 2015). Utilising a comprehensive and validated framework like the CASI classification tool can help reduce classification confusion, enhance diagnostic accuracy and guide clinical decision‐making in the subsequent treatment of and prevention of these injuries. The introduction of the new aetiological category, complex clinical presentation, recognises and addresses the evidence and clinical practice gap regarding the potential complexity in the classification, treatment and prevention of CASI. Further research using a larger group of potential users of this instrument is necessary to conduct extensive validity and reliability testing.

## 7 Conclusion

Our study represents a significant step forward in CASI classification and management using a modified Delphi method. Until now, a validated CASI classification tool has been lacking. Our study addresses this gap by proposing a comprehensive and validated classification tool to support clinical decision‐making and to provide a common language for healthcare providers, educators and researchers. The availability of an effective classification system can reduce clinical uncertainty, improve treatment and prevention and improve patient outcomes. Further research is warranted to investigate the use of this system in clinical practice.

## Ethics Statement

The study has received ethical approval from the Children's Health Queensland Hospital and Health Service Human Research Ethics Committee (HREC/15/QRCH/241) and Griffith University Ethics Committee (2016/063).

## Conflicts of Interest

The authors declare no conflicts of interest.

### Peer Review

The peer review history for this article is available at https://www.webofscience.com/api/gateway/wos/peer‐review/10.1111/jan.16416.

## Supporting information


Table S1.

## Data Availability

The data that support the findings of this study are available on request from the corresponding author. The data are not publicly available due to privacy or ethical restrictions.
